# Astaxanthin Overproduction Enhanced by Metabolomics-Guided Rational Metabolic Engineering in *Synechococcus* sp. PCC 7002

**DOI:** 10.1021/acssynbio.5c00490

**Published:** 2025-11-10

**Authors:** Kousuke Ida, Kenya Tanaka, Yuichi Kato, Nobuaki Koike, Yoji Horie, Mami Matsuda, Hisashi Yasueda, Akihiko Kondo, Tomohisa Hasunuma

**Affiliations:** † 13447 Kawasaki Frontience R&D Center, Toagosei Co., Ltd., 3-25-40 Tonomachi, Kawasaki, Kanagawa 210-0821, Japan; ‡ Engineering Biology Research Center, 12885 Kobe University, 1-1 Rokkodai, Nada, Kobe 657-8501, Japan; § Graduate School of Science, 592891 Innovation and Technology, Kobe University, 1-1 Rokkodai, Nada, Kobe 657-8501, Japan; ∥ Research Center for Solar Energy Chemistry, Graduate School of Engineering Science, The University of Osaka, Toyonaka, Osaka 560-8531, Japan; ⊥ Department of Biotechnology, Toyama Prefectural University, 5180 Kurokawa, Imizu, Toyama 939-0398, Japan; # RIKEN Center for Sustainable Resource Science, 1-7-22 Suehiro, Tsurumi, Yokohama, Kanagawa 230-0045, Japan; ∇ Department of Chemical Science and Engineering, Graduate School of Engineering, Kobe University, 1-1 Rokkodai, Nada, Kobe 657-8501, Japan; ○ Research and Development Center for Precision Medicine, University of Tsukuba, 1-2 Kasuga, Tsukuba-Shi, Ibaraki 305-8550, Japan

**Keywords:** astaxanthin, metabolomics, cyanobacteria, transketolase, carotenoid, design-build-test-learn (DBTL)

## Abstract

Astaxanthin, a natural red pigment with antioxidant and other physiological activities, is widely used in the feed, pharmaceutical, and cosmetic industries. *Haematococcus pluvialis* is a well-known microbial producer of astaxanthin; however, its slow growth and requirement for a complex two-stage cultivation under high-light conditions limit large-scale application due to increased contamination risk. As an alternative, *Synechococcus* sp. PCC 7002 offers rapid growth and robustness, but metabolic engineering strategies to enhance astaxanthin production in this strain remain underexplored. In this study, we applied a metabolomics-guided approach to identify novel metabolic bottlenecks and engineering targets. A base strain expressing β-carotene hydroxylase (*crtZ*) and ketolase (*crtW*) from *Brevundimonas* sp. SD212 produced 6.2 mg/g of DCW astaxanthin. Metabolome analysis revealed the accumulation of sedoheptulose-7-phosphate (S7P) and 2-C-methyl-d-erythritol-2,4-cyclopyrophosphate (MEcPP), suggesting that the reactions catalyzed by transketolase (TKT) and (E)-4-hydroxy-3-methyl-but-2-enyl pyrophosphate synthase (IspG) are rate-limiting. Overexpression of *tkt* or *ispG* reduced the levels of their respective substrates, confirming relief of these bottlenecks. Notably, the TKT-overexpressing strain achieved an astaxanthin content of 10.3 mg/g-DCW, while the IspG-overexpressing strain showed no significant improvement. Further optimization of culture conditions—such as medium composition, light intensity, and temperature—led to an astaxanthin productivity of 7.5 mg/L/day. These results demonstrate the effectiveness of a metabolomics-driven design-build-test-learn (DBTL) approach for enhancing astaxanthin production in *Synechococcus* sp. PCC 7002.

## Graphical abstract

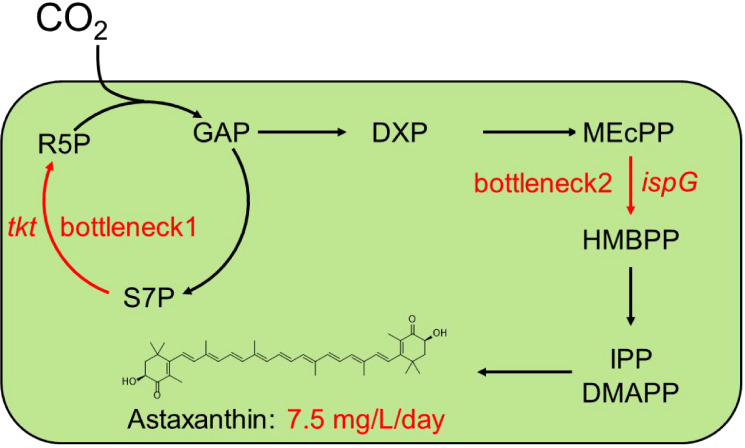

## Introduction

Astaxanthin (3,3′-dihydroxy-β,β-carotene-4,4’-dione) is a red pigment found in the shells of crustaceans such as shrimp and crabs. It is a β-carotene derivative, modified by 3,3′-dihydroxylation and 4,4’-diketonization. Astaxanthin not only possesses singlet oxygen-quenching abilities approximately 40 times higher than those of β-carotene,^1^ but also exhibits a range of physiological activities, including antitumor properties and the prevention of skin aging.^2–4^ Recently, in addition to its use in animal feed, astaxanthin has been increasingly utilized in the food and cosmetics industries.

Astaxanthin currently available on the market includes synthetic variants; however, while naturally derived astaxanthin exists as the stereoisomers (3S, 3′S) or (3R, 3′R), synthetic astaxanthin contains a mixture of (3S, 3′S), (3R, 3′S), and (3R, 3′R) in a 1:2:1 ratio, resulting in the production of non-natural forms of astaxanthin. The antioxidant activity of the synthetic product is lower compared to its natural counterpart, leading to a preference for naturally derived astaxanthin in applications where high antioxidant capacity is essential.^5^

Natural astaxanthin production methods have been established using green algae, yeast, and bacteria.^6–8^ Among these, the freshwater green alga *Haematococcus pluvialis* is widely used, not only due to its high astaxanthin productivity but also because it can grow using CO_2_ as a carbon source.^6^ *H. pluvialis* can accumulate astaxanthin up to approximately 4% of its dry cell weight by converting from the “green stage,” where the cells proliferate, to the “red stage,” where astaxanthin production is induced under high-stress conditions during the culture process. However, this two-stage process requires a complicated procedure and long cultivation periods (over 10 days). Furthermore, since the green stage thrives under low-temperature and low-light conditions, maintaining these optimal culture conditions throughout outdoor cultivation would be a major challenge. In addition, the large-scale freshwater cultivation system poses a risk of contamination. During the red stage, astaxanthin accumulation is induced by various stress conditions, including high light intensity, nitrogen deprivation, and salt stress. However, the precise mechanisms by which each stressor induces astaxanthin biosynthesis remain incompletely understood, limiting efforts to optimize cultivation conditions.^9^

Alternative host organisms have the potential to solve the problems of astaxanthin production using *H. pluvialis*. While many microalgae possess the metabolic pathways necessary for synthesizing β-carotene, the conversion of β-carotene to astaxanthin requires specific β-carotene hydroxylase (CrtZ) and β-carotene ketolase (CrtW) enzymes with appropriate substrate specificities (Figure 1). The activity and substrate specificity of CrtZ and CrtW can vary significantly among enzymes. Some CrtZ enzymes preferentially hydroxylate β-carotene, while others may act more efficiently on intermediate carotenoids such as echinenone or canthaxanthin. Similarly, CrtW enzymes differ in their ability to introduce keto groups at specific positions on different substrates. As a result, even if a host microalga expresses both *crtZ* and *crtW* genes, it does not necessarily guarantee efficient conversion of β-carotene to astaxanthin. Thus, introducing specific *crtZ* and *crtW* genes into host microalgae has enabled the development of strains capable of astaxanthin production (Table 1). For example, the introduction of *crtZ* and *crtW* from *Brevundimonas* sp. SD-212 into *Synechocystis* sp. PCC 6803 resulted in an astaxanthin accumulation of 1.1 mg/g-DCW.^10^

**Table 1:** Recent Advances in Astaxanthin Biosynthesis Using Genetically Modified Microalgae

	Halotolerance	Cultivation time (d)	Content (mg/g- DCW)	Productivity (mg/L/day)	references
*Synechocystis* sp.Pcc 6803	Freshwater	-	1.10	-	^11^
*Synechocystis* sp.Pcc 6803	Freshwater	10	29.6	2.96	^12^
*Chlamydomonas reinhardtii*	Freshwater	7	-	3.10	^13^
*Dunaliella viridis*	Halotolerant	-	0.08	-	^15^
*Synechococcus* sp. PCC7002	Halotolerant	2	3.00	3.35	^17^
*Synechococcus* sp. PCC 11901	Halotolerant	-	5.9	9.6	^42^
*Synechococcus* sp. PCC 7002	Halotolerant	7	11.8	7.5	This study

**Figure 1:**
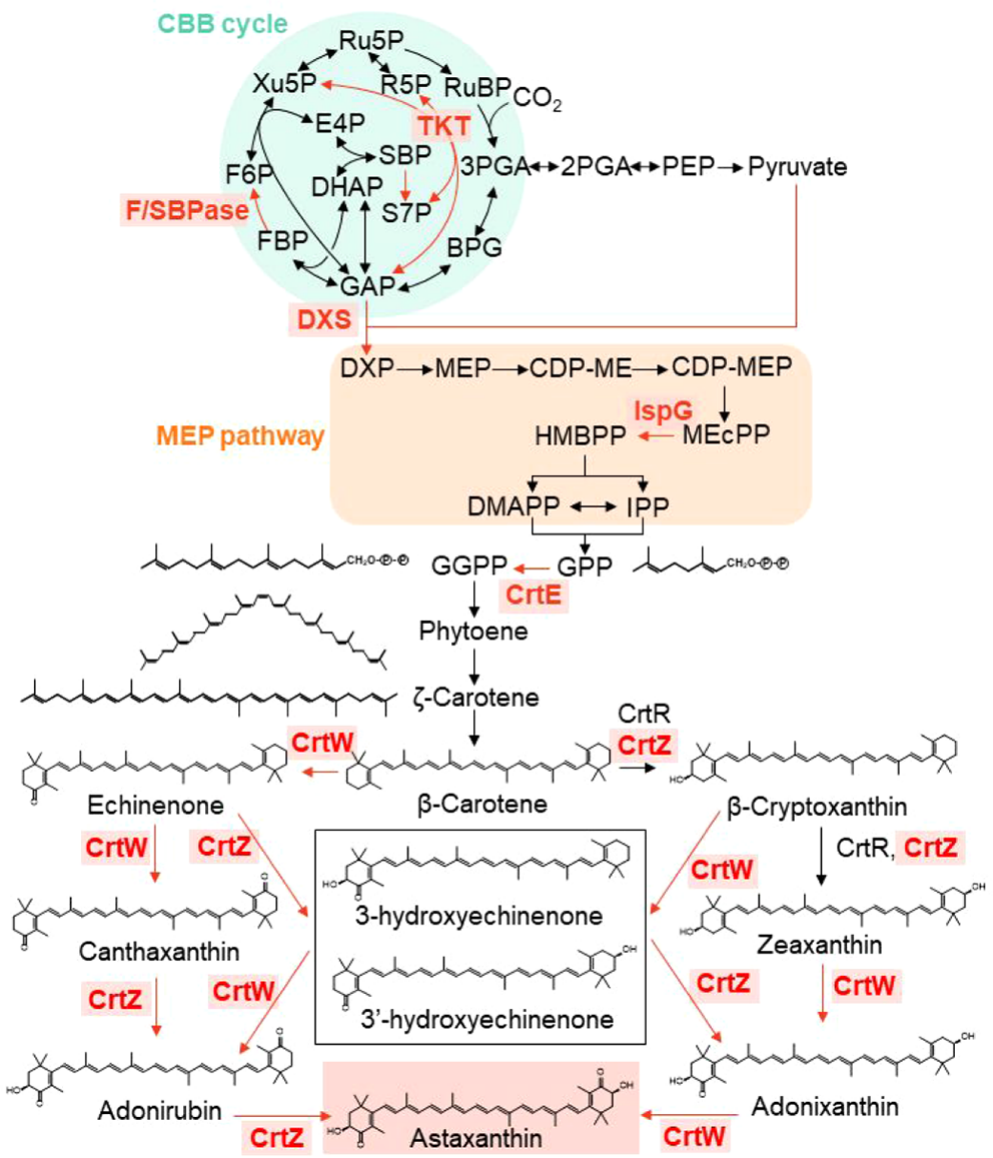
Biosynthesis pathway to astaxanthin. Enzymes are indicated by their gene symbols. Enzymes introduced or overexpressed in this study are highlighted in bold red letters within the shallow red square. Abbreviations: BPG, 1,3-bisphosphoglycerate; CDP-ME, 4-diphosphocytidyl-2-C-methylerythritol; CDP-MEP, 4-diphosphocytidyl-2-C-methyl-d-erythritol 2-phosphate; CrtE, geranylgeranyl diphosphate synthase; CrtR, β-carotene hydroxylase; CrtW, β-carotene ketolase; CrtZ, β-carotene hydroxylase; DHAP, dihydroxyacetone phosphate; DMAPP, dimethylallyl diphosphate; DXP, 1-deoxy-d-xylulose 5-phosphate; DXS, 1-deoxy-d-xylulose 5-phosphate synthase; E4P, erythrose 4-phosphate; FBP, fructose 1,6-bisphosphate; F6P, fructose 6-phosphate; F/SBPase, fructose 1,6-/sedoheptulose 1,7-bisphosphatase; GAP; glyceraldehyde 3-phosphate; GGPP, geranylgeranyl diphosphate; GPP, geranyl diphosphate; HMBPP, 4-hydroxy-3-methyl-2-(*E*)-butenyl diphosphate; IPP, isopentenyl pyrophosphate; IspD, 2-C-methyl-d-erythritol 4-phosphate cytidylyltransferase; IspE, 4-diphosphocytidyl-2-C*-*methyl-d-erythritol kinase; IspG, 1-hydroxy-2-methyl-2-(*E*)-butenyl 4-diphosphate synthase; IspH, 4-hydroxy-3-methylbut-2-enyl diphosphate reductase; MEcPP, 2-C-methyl-d-erythritol-2,4-cyclopyrophosphate; MEP, 2-C-methyl-d-erythritol 4-phosphate; PEP, phosphoenolpyruvate; PGK, phosphoglycerate kinase; PRK, phosphoribulokinase; R5P, ribose 5-phosphate; Ru5P, ribulose 5-phosphate; SBP, sedoheptulose 1,7-bisphosphate; RuBP, ribulose 1,5-bisphosphate;S7P, sedoheptulose 7-phosphate; TKT, transketolase; Xu5P, xylulose 5-phosphate; 2PGA, 2-phosphoglyceric acid; 3PGA, 3-phosphoglyceric acid.

Moreover, to improve astaxanthin productivity, various metabolic engineering approaches have been explored.^11^ In *Synechocystis*, coexpression of genes encoding fructose-1,6-/sedoheptulose-1,7-bisphosphatase (F/SBPase), 1-deoxy-d-xylulose-5-phosphate synthase (DXS), and farnesyl diphosphate synthase (IspA), along with *crtZ* and *crtW* genes, increased astaxanthin productivity to 29.9 mg/g-DCW and 2.8 mg/L/day.^12^ Other examples of freshwater microalgae that have been genetically engineered for astaxanthin production include *Chlamydomonas reinhardtii* and *Dunaliella viridis*, though their astaxanthin productivity remains relatively low (Table 1).^13–15^

To reduce contamination risks during cultivation, halotolerant strains are advantageous.^16^ The halophilic cyanobacterium *Synechococcus* sp. PCC 7002, with a doubling time of ∼2.5 h and the ability to grow in high-salinity conditions (e.g., 0.5 M NaCl), represents a promising host for astaxanthin production. Previously, we achieved astaxanthin production of 3 mg/g-DCW and 3.35 mg/L/day by expressing *crtZ* and *crtW* on a multicopy plasmid in this strain.^17^ However, studies of astaxanthin biosynthesis in *Synechococcus* sp. PCC 7002 remain limited, and rationally identified metabolic engineering targets for improving production are still lacking.

Design–Build–Test–Learn (DBTL) cycle is a powerful research framework for constructing high-producing microbial strains from the base strains in a short working term.^18^ In this DBTL-cycle workflow, metabolomics plays a crucial role in the test (T) phase, enabling detailed strain evaluation and facilitating the identification of metabolic bottlenecks, which inform subsequent design (D) improvements.^19,20^ This workflow has been successfully applied to strain development for the production of various compounds, such as acetate, polyhydroxybutyrate, isobutyraldehyde, 2,3-butanediol, d-lactate, and succinate in microalgae and cyanobacteria.^21^ One study used metabolomics to guide engineering for astaxanthin production in *Synechocystis* PCC 6803.^12^

In this study, we constructed an astaxanthin-producing base strain of *Synechococcus* sp. PCC 7002 by introducing *crtZ* and *crtW* from *Brevundimonas* sp. SD212. To further enhance astaxanthin productivity, we introduced additional enzymes that have previously been reported to catalyze rate-limiting steps in the biosynthesis of astaxanthin and related compounds. To identify further metabolic engineering targets specific to *Synechococcus* sp. PCC 7002, we performed a comprehensive metabolome analysis of the base strain to pinpoint metabolic bottlenecks. Based on the metabolomic data, we conducted systematic strain engineering to relieve these bottlenecks. As a result, we confirmed that the overexpression of genes selected through metabolomic analysis effectively alleviated the identified pathway limitations. Finally, astaxanthin productivity was further improved under high-cell-density cultivation conditions.

## Results

### Astaxanthin Production by *Synechococcus* sp. PCC 7002 Cells Integrated with Heterologous *crtZ* and *crtW* Genes

*Synechococcus* sp. PCC 7002 naturally produces β-carotene and various xanthophylls, including zeaxanthin and 3′-hydroxyechinenone.^22^ Astaxanthin biosynthesis requires the addition of ketone and hydroxyl groups to β-carotene, catalyzed by CrtZ and CrtW. However, wild-type (WT) *Synechococcus* sp. PCC 7002 does not produce astaxanthin, likely due to the low efficiency of its ketolase in converting zeaxanthin to astaxanthin via adonixanthin.^23,24^ To overcome this limitation, we previously introduced the *crtZ* and *crtW* genes from *Brevundimonas* sp. SD212, whose products (enzymes) have shown superior catalytic activity in *Synechococcus* sp. PCC 7002.^17^ *Brevundimonas* CrtW efficiently converts adonixanthin to astaxanthin,^25^ and its CrtZ exhibits higher β-carotene hydroxylation efficiency than homologus from other bacteria.^26^

While the *crtZ* and *crtW* genes from *Brevundimonas* sp. SD212 were previously expressed from an endogenous plasmid;^17^ we constructed a strain named Strain 1 by integrating both genes as an operon under the strong native promoter *psbA2* into the *glpK* site of the chromosome in the WT *Synechococcus* sp. PCC 7002. The *glpK* site was chosen as a neutral insertion site for the introduction of genes since *glpK* is a pseudogene in this strain due to a frameshift mutation (Figure 2a).^27^ The successful gene insertion at the target locus was confirmed by the electrophoresis analysis shown in Figure 2b.

**Figure 2:**
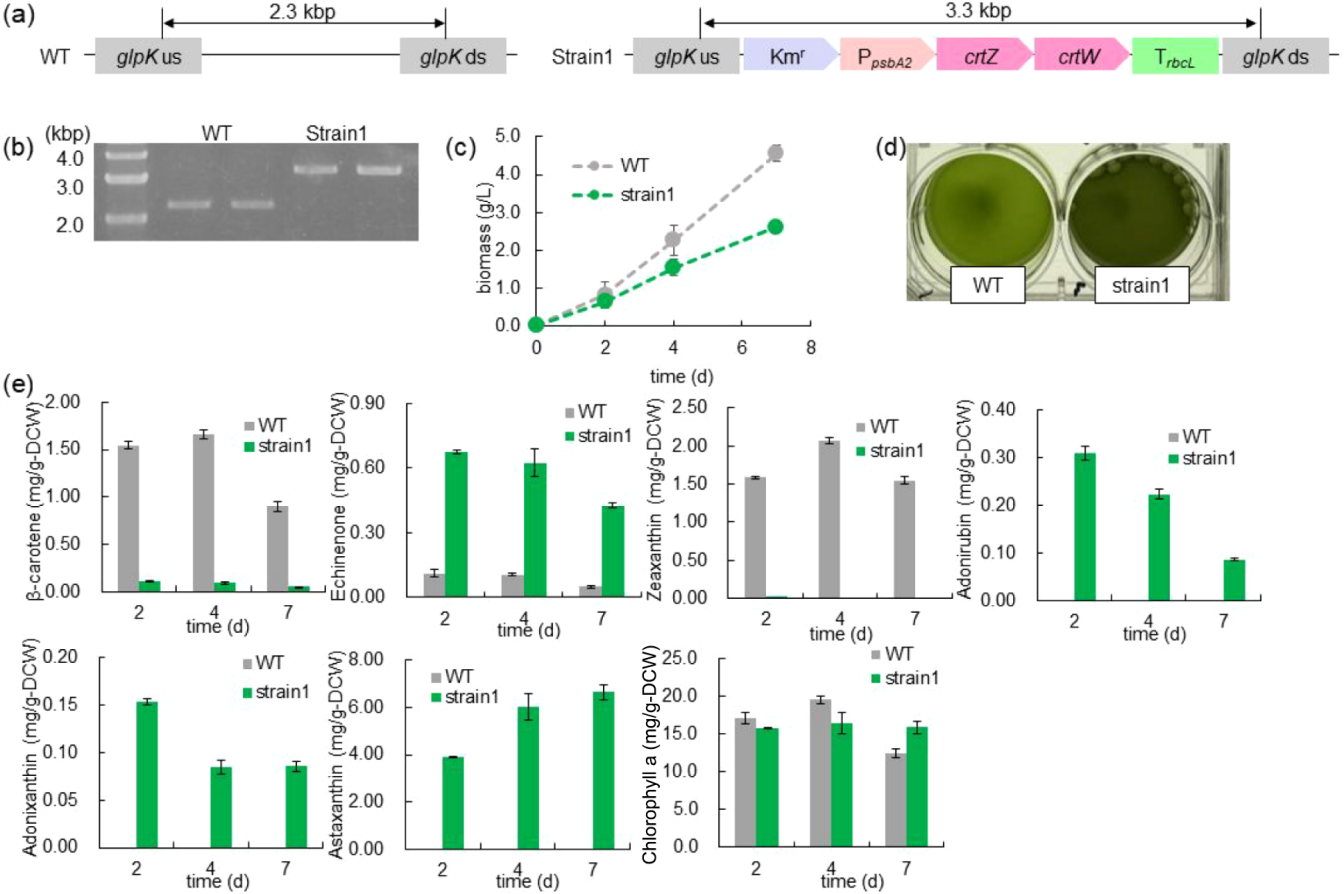
Construction of the *crtZ/crtW* homologous recombination strain (strain1) and evaluation of pigment production. (a) Schematic representation of the homologous recombination sites in the genomes of wild type (WT) and strain1. Km^r^, kanamycin resistance cassette; P_*psbA2*_, *psbA2* promoter; *crtZ*, β-carotene hydroxylase from *Brevundimonas* sp. SD212; *crtW*, β-carotene ketolase from *Brevundimonas* sp. SD212; T_*rbcL*_, *rbcL* terminator. (b) PCR analysis of the homologous recombination regions in WT and Strain 1. (c) Time course of cellular biomass accumulation. (d) Visible color difference in the culture medium between WT and Strain 1 on day 7 of cultivation. (e) Time course of pigment production in the *crtZ/crtW*-expressing Strain 1 (green) compared to WT (gray) when cultured for 7 days under 100 μmol m^–2^ s^–1^ light intensity and 2% CO_2_. Values represent the mean ± SE of three biological replicates.

The production of pigments, including β-carotene and its derivatives, in the WT strain and Strain 1 was evaluated. Cultures were grown under 100 μmol/m^2^/s light intensity, 2% (v/v) CO_2_, and at 30 °C. Figure 2c shows the growth profiles of both strains during cultivation. After 7 days, the WT reached 3.9 g DCW/L, whereas Strain 1 reached 2.1 g DCW/L, indicating that the gene insertion negatively affected growth. However, as seen in Figure 2d, the culture medium of Strain 1 after cultivation had a dark green color compared to that of the wild-type strain, suggesting an alteration in pigment production of Strain 1. Next, we performed quantitative analysis of the carotenoids present within the cells. The pigments were quantified using ultraperformance liquid chromatography-photodiode array (UPLC-PDA), and the results are shown in Figure 2e. In the WT strain, zeaxanthin and β-carotene were the primary carotenoids accumulated in the cells. In Strain 1, intracellular accumulation of these major carotenoids was reduced compared to that in the strain WT, while adonirubin and adonixanthin were newly found in Strain 1. Astaxanthin was produced as the dominant pigment in Strain 1, and the accumulation of astaxanthin reached 6.63 mg/g-DCW. Thus, we constructed Strain 1 as a basic strain for astaxanthin production from *Synechococcus* sp. PCC7002.

### Effect of Overexpression of *dxs, crtE,* or *f/sbp* Gene on Astaxanthin Production

To further improve astaxanthin productivity, we evaluated three genes previously reported to enhance isoprenoid or carotenoid production. Since isoprenoids are synthesized from the same precursor as carotenoids, geranylgeranyl diphosphate (GGPP), metabolic engineering strategies that enhance the production of GGPP for isoprenoid overproduction are also likely to be effective for boosting carotenoid production. A previous study demonstrated that the introduction of the *dxs* gene from *Deinococcus radiodurans* into *Synechocystis* PCC 6803 successfully improved isoprene production.^28^ Due to its decarboxylation reaction, DXS provides a strong thermodynamic driving force and, as the first committed step of the MEP pathway, plays a critical role in determining the influx into this pathway. To examine whether similar effects could be observed in *Synechococcus* sp. PCC 7002, we constructed and evaluated Strain 2, which expresses the *dxs* gene from *D. radiodurans*. We also constructed Strain 3, overexpressing the endogenous *crtE* gene, which encodes geranylgeranyl diphosphate synthase (CrtE), a key enzyme in the MEP pathway known to enhance limonene productivity in *Synechocystis* PCC 6803.^29^ Additionally, we developed Strain 4, which overexpresses the endogenous *f/sbp* gene. This gene encodes F/SBPase, which is considered a rate-limiting enzyme in the Calvin cycle of cyanobacteria, and whose overexpression has previously been shown to enhance photosynthetic capacity.^30,31^ Then, each gene (*dxs, crtE,* and *f/sbp*) was expressed under the control of either the *psbA2* promoter or another endogenous strong promoter, *rbcL* (Figure 3a). Each construct was integrated into a different chromosomal locus—*acsA, *A1202*, or *A0936*—on the chromosome of Strain 1 (Figure 3a). The *A1202* site and *A0936* site can function as neutral sites for genome integration in *Synechococcus* sp. PCC 7002.^32,33^ The *acsA* site can also be used for genome integration without any growth defect.^27^

**Figure 3:**
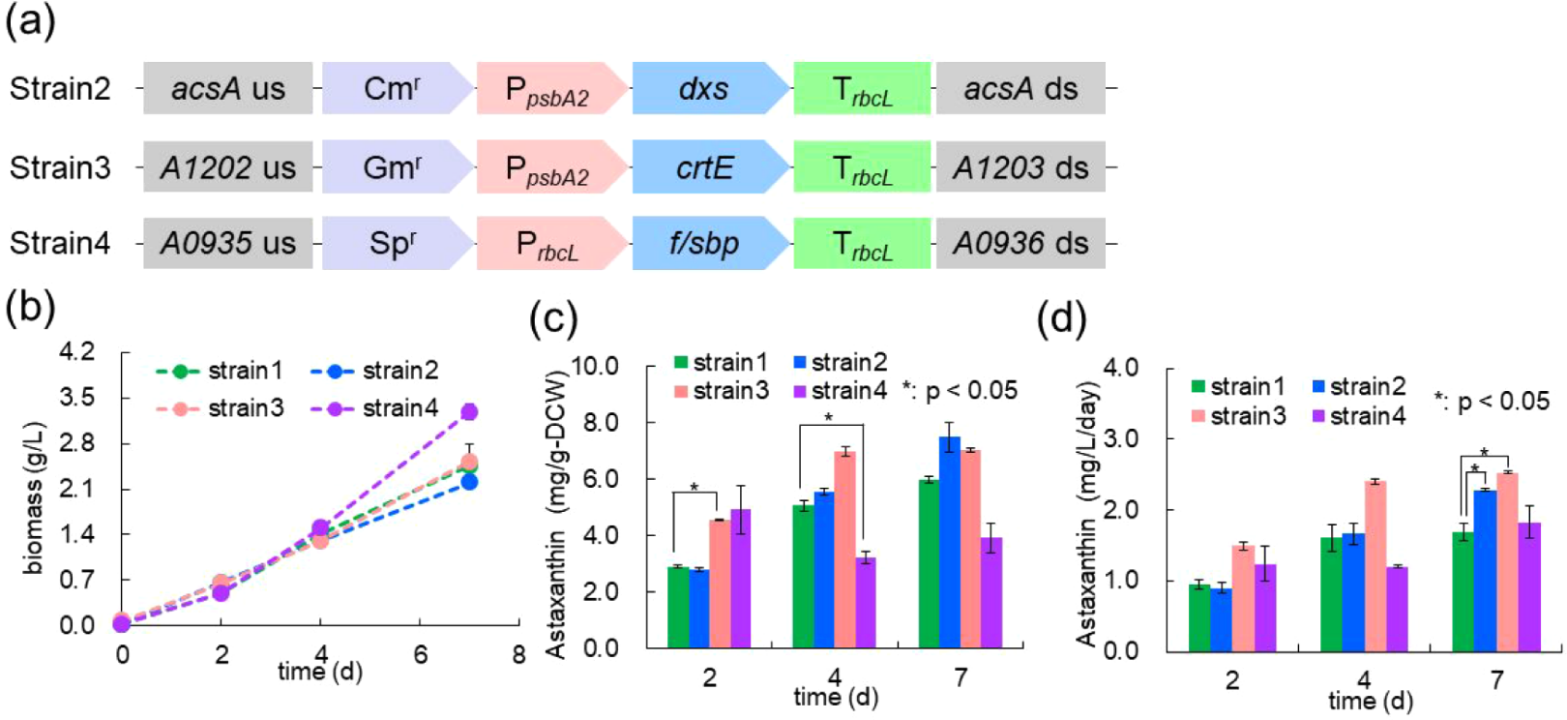
Effect of the overexpression of genes in the Calvin cycle and MEP pathway based on previous studies: (a) Schematic images of target gene insertion at the homologous recombination site. *Cm* ^r^, chloramphenicol resistance cassette; P_*psbA2*_, *PsbA2* promoter; *dxs*, 1-deoxy-d-xylulose 5-phosphate synthase from *Deinococcus radiodurans*; T_*rbcL*_, *rbcL* terminator; Gm^r^, gentamicin resistance cassette; *crtE*, geranylgeranyl diphosphate synthase from *Synechococcus* sp. PCC 7002; Sp^r^, *Spectinomycin* resistance cassette; *f*/*sbp*, fructose 1,6-/sedoheptulose 1,7-bisphosphatase from *Synechococcus* sp. PCC 7002. (b) Time course of biomass yield corresponding to the expression of each gene. (c) Comparison of astaxanthin content among Strains 1 to 4. (d) Effect of genetic engineering on daily astaxanthin productivity. Values represent the average (±SE) of two biological replicates. Statistical significance was determined using an unpaired *t*-test (*: *p* < 0.05).

Strains 2 and 3 exhibited growth profiles similar to Strain 1, while Strain 4 showed improved growth after 4 days of cultivation (Figure 3b). The astaxanthin content of each strain is shown in Figure 3c. In Strain 2, astaxanthin levels remained unchanged compared to Strain 1 across all time points. In Strain 3, the astaxanthin content increased to approximately 1.6-fold that of Strain 1 at day 2, reaching 4.6 mg/g-DCW. In contrast, Strain 4 showed a decrease in astaxanthin content after day 4 of cultivation. Astaxanthin productivity increased in both Strains 2 and 3, with Strain 3 reaching a maximum of 2.5 mg/L/day (Figure 3d). Other pigment profiling for Strains 1–4 showed that, in the strains with improved astaxanthin metrics (Strains 2 and 3), adonixanthin, echinenone, and β-carotene tended to increase relative to Strain 1 at selected time points (SuppFigure S1). These results demonstrate that overexpression of *dxs* or *crtE* in the astaxanthin-producing base strain (Strain 1) contributes to enhanced productivity.

However, overexpression of the *f/sbp* gene was effective for enhancing astaxanthin production in *Synechocystis* sp. PCC 6803;^12^ it had the opposite effect in Strain 1 derived from *Synechococcus* sp. PCC 7002. These findings suggest that strain-specific strategies are necessary for improving astaxanthin production in *Synechococcus* sp. PCC 7002. Although Strain 3 achieved improved productivity (2.5 mg/L/day), it still fell short of the levels reported in earlier studies (Table 1), underscoring the need to identify additional metabolic engineering targets. Based on these observations, we conducted further metabolomic analyses to uncover previously unrecognized bottleneck reactions and to select new gene targets for rational strain improvement.

### Metabolome Analysis of Astaxanthin-Producing Cells

To identify bottleneck reactions in astaxanthin biosynthesis, we first conducted a metabolome analysis comparing the WT with Strain 1, which serves as the foundational astaxanthin-producing strain harboring *crtZ* and *crtW*. This comparison was intended to reveal how the introduction of these key astaxanthin biosynthetic genes alters the central metabolic pathways relative to the unmodified background. Understanding these baseline changes was essential before analyzing further engineered strains, as it allowed us to pinpoint initial pathway limitations directly associated with astaxanthin biosynthesis. Therefore, Strain 1 was selected as the starting point for a detailed metabolomic analysis.

We quantified intracellular metabolite pool sizes in both the WT and Strain 1. Our analysis focused on central carbon metabolism and isoprenoid precursor pathways to identify the potential bottleneck reaction for carotenoid synthesis. Among glycolytic intermediates, no major differences were observed between strains (Figure 4). However, Strain 1 showed elevated levels of sedoheptulose-7-phosphate (S7P) on day 7, and both strains exhibited relatively high accumulation of MEcPP in the MEP pathway on days 2 and 4.

**Figure 4:**
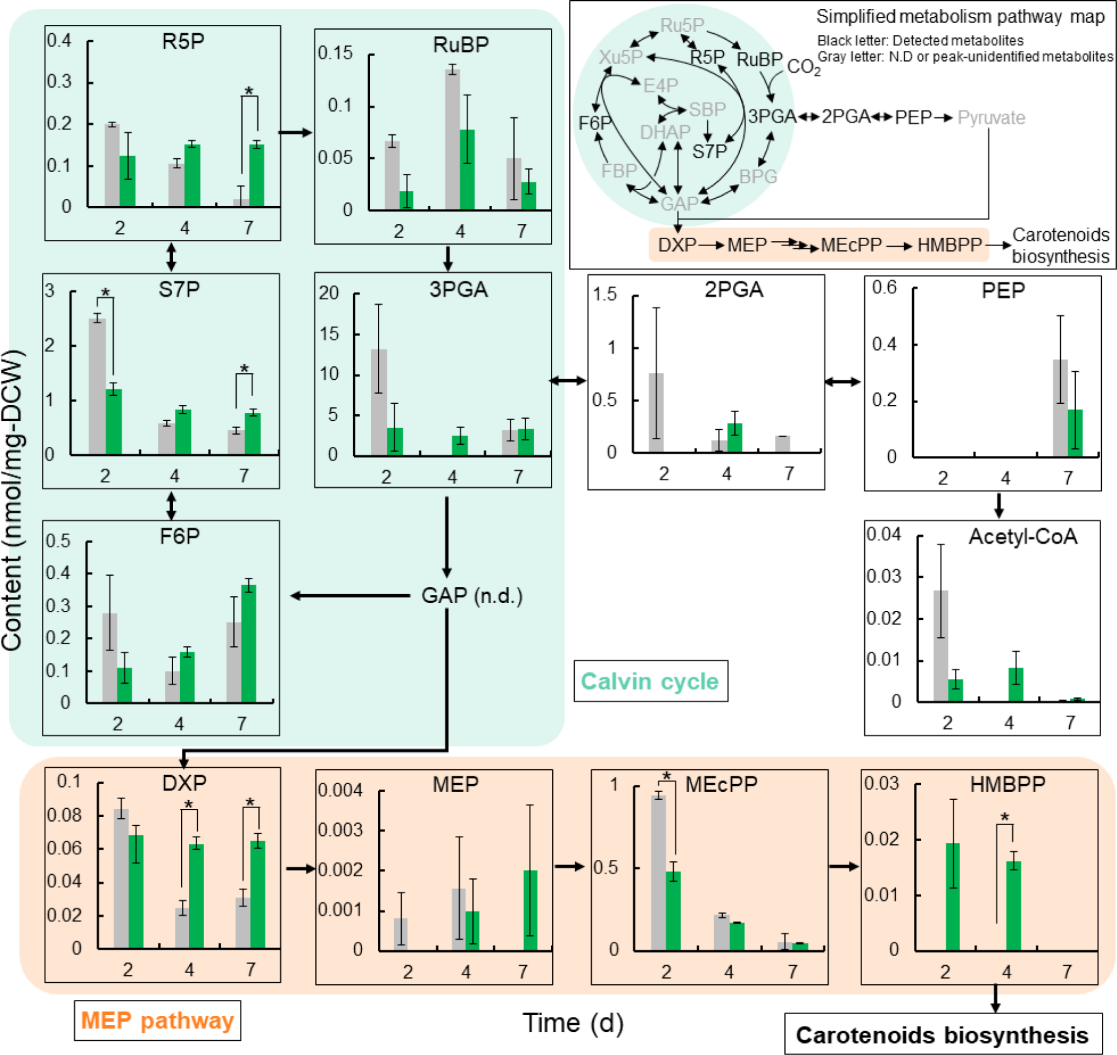
Metabolome analysis of the wild type (gray bar) and Strain 1 (green bar). Values represent the average (±SE) of three biological replicates. Statistical significance was determined using an unpaired *t*-test (*: *p* < 0.05). n.d., not detected.

### Enhancement of Astaxanthin Production by Relieving the Bottleneck on the Metabolic Flows

Based on the metabolome analysis, it was suggested that the slow conversion of sedoheptulose-7-phosphate (S7P) to ribose-5-phosphate (R5P) and of MEcPP to HMBPP may represent metabolic bottlenecks limiting astaxanthin production in Strain 1. To alleviate these constraints, we constructed two new strains derived from Strain 1, each overexpressing one of the enzymes responsible for the putative bottleneck steps: transketolase (TKT), encoded by the endogenous *tkt* gene, and HMBPP synthase (IspG), encoded by the endogenous *ispG* gene (Figure 5a). The *tkt* gene was integrated into the *acsA* locus to generate the TKT-overexpressing strain, designated Strain 5. The *ispG* gene was inserted into the *A0026* site, a chromosomal region between two putative neutral genes previously identified as a suitable integration site,^34^ to construct the IspG-overexpressing strain, designated Strain 6.

**Figure 5:**
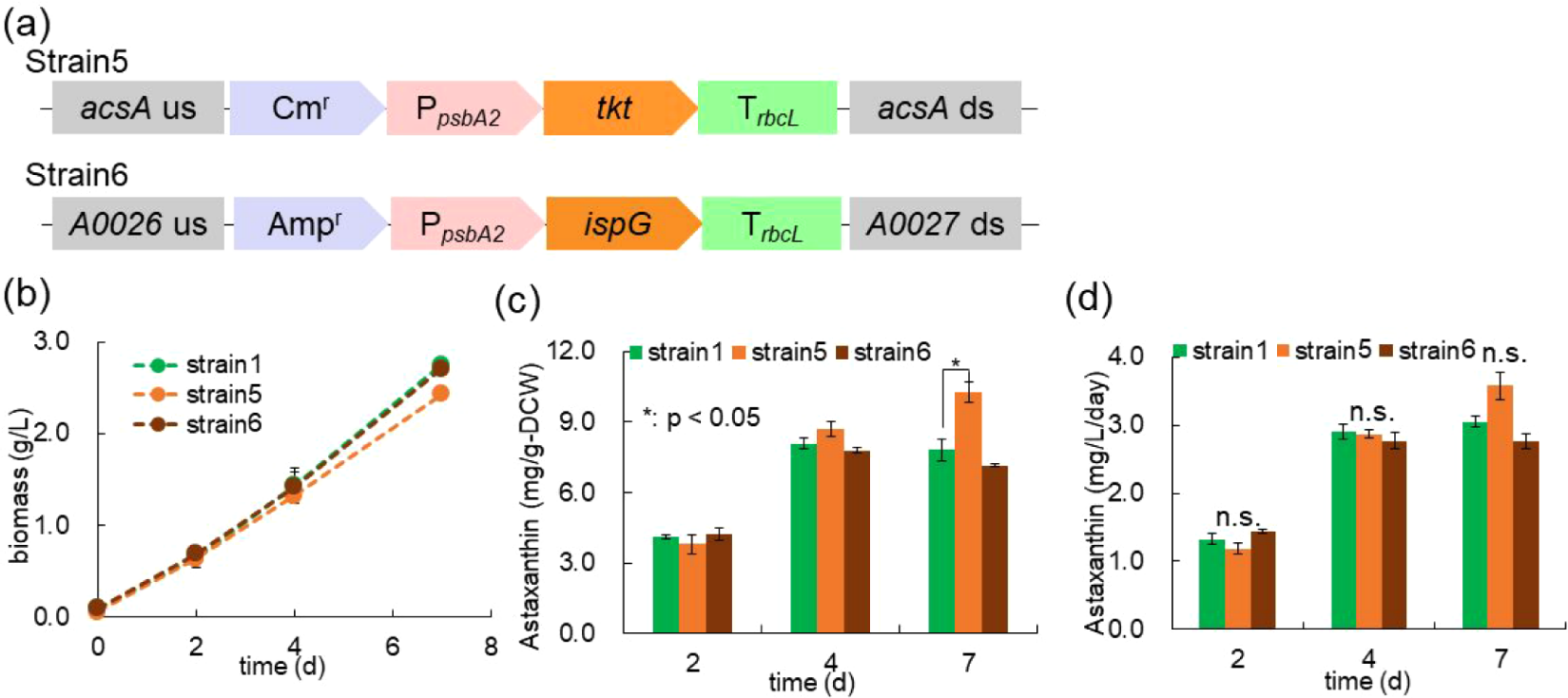
Effect of overexpression of endogenous genes encoding transketolase (*tkt*) and 1-hydroxy-2-methyl-2-(*E*)-butenyl 4-diphosphate synthase (*ispG*) on astaxanthin production. (a) Schematic of target gene insertion at the homologous recombination site. (b) Time course of cellular biomass. (c,d) Astaxanthin cellular content and productivity of engineered strains. Values represent the average (±SE) of three biological replicates. Statistical significance was determined using *t*-test (*: *p* < 0.05). n.s., not significant.

Strain 5 exhibited a slight reduction in biomass compared to Strain 1, while Strain 6 showed a growth profile similar to that of Strain 1 (Figure 5b). After 7 days of cultivation, the biomass of Strain 5 and Strain 6 reached 2.5 g/L and 2.7 g/L, respectively. The astaxanthin content in Strain 5 increased to 10.3 mg/g-DCW, whereas Strain 6 showed no notable change in the astaxanthin content relative to Strain 1 (Figure 5c). Despite the increased astaxanthin content, Strain 5 did not exhibit a significant improvement in overall astaxanthin productivity, likely due to its slightly lower biomass (Figure 5d).

To verify whether the identified metabolic bottlenecks were alleviated in Strains 5 and 6, we performed an additional round of metabolome analysis. This analysis focused on the substrates and products of the enzymatic reactions catalyzed by TKT and IspG. In Strain 5, the intracellular level of S7P—the substrate of TKT—was reduced compared to Strain 1 on both day 2 and day 4 of cultivation (Figure 6a). Similarly, in Strain 6, the concentration of MEcPP—the substrate of IspG—was lower than that in Strain 1 and fell below the detection limit of the assay (Figure 6b). These results indicate that overexpression of the target genes effectively relieved the metabolic bottlenecks previously identified in Strain 1. Moreover, since Strain 5 exhibited an increase in the astaxanthin content, we concluded that the genetic modification targeting *tkt* functioned as intended.

**Figure 6:**
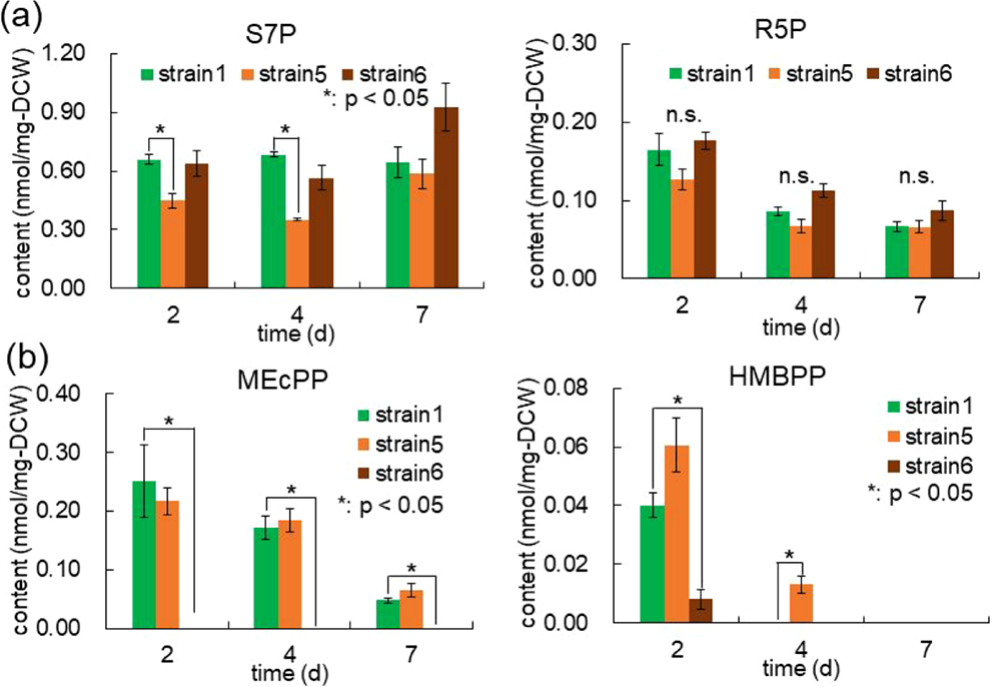
Metabolome analysis of *Synechococcus* sp. PCC 7002 strains overexpressing *tkt* or *ispG*. Concentrations of the substrate and the product of Tkt (a) or IspG (b) in cells upon *tkt* overexpression or *ispG* overexpression. Values represent the average (±SE) of three independent experiments. Statistical significance was determined using an unpaired *t*-test (*: *p* < 0.05). n.s., not significant.

### Production of Astaxanthin by *Synechococcus* sp. PCC 7002 under Improved Culture Conditions

Previous studies have reported that cyanobacteria achieve higher biomass yields when cultured in MAD2 medium under high light intensity conditions.^35,36^ To evaluate whether the engineered astaxanthin-producing strains (Strains 1 and 5) would similarly benefit from such conditions, we compared their performance under two defined culture conditions, referred to as Conditions A and B. Condition A served as the standard cultivation condition: cells were grown in MA2 medium, starting at an initial optical density at 750 nm (OD_750_) of 0.1, and incubated at 30 °C under 2% (v/v) CO_2_, illuminated with white light at 100 μmol/m^2^/s, and agitated at 100 rpm for 7 days. In contrast, Condition B was designed as a high-cell-density cultivation condition: cultures were initiated in MAD2 medium at an initial OD_750_ of 0.5 and grown overnight at 38 °C under 5% (v/v) CO_2_ and 150 μmol/m^2^/s white light while being shaken at 150 rpm. After the first day, the light intensity was increased to 500 μmol/m^2^/s, and cultivation was continued for an additional 6 days. Under Condition B, Strain 1 exhibited a marked improvement in biomass accumulation, with maximum dry cell weight reaching 4.9 g/L—approximately double that observed under Condition A (Figure 7a). This result demonstrates that optimization of culture parameters can significantly enhance the biomass yield, which may contribute to improved astaxanthin productivity. In addition, the astaxanthin content in Strain 1 was significantly increased after 7 days of cultivation under Condition B (Figure 7b).

**Figure 7:**
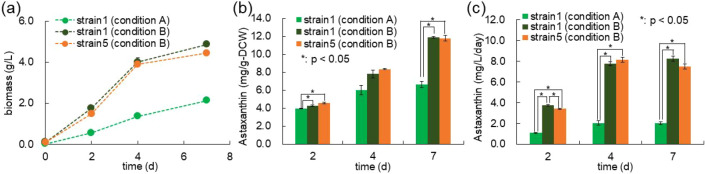
Optimization of pigment biosynthesis through alterations in cultivation conditions. (a) Comparison of biomass time course following the improvement of culture conditions. (b) Astaxanthin production under high-density conditions. (c) Comparison of astaxanthin productivity under conventional and high-density cultivation conditions. Condition A was defined as A2 medium, 100 μmol/m^2^/s light intensity, 2% CO_2_, 30 °C, and 100 rpm. Condition B was established as MAD2 medium, 500 μmol/m^2^/s light intensity, 5% CO_2_, 38 °C, and 150 rpm. Values represent the average (±SE) of three biological replicates. Statistical significance was determined using an unpaired *t*-test (*: *p* < 0.05).

To further enhance astaxanthin accumulation, we evaluated Strain 5 under the same conditions, as it had exhibited the highest productivity under Condition A (Figure 5). Although the biomass of Strain 5 was slightly lower than that of Strain 1 on day 7 (Figure 7a), its astaxanthin content increased markedly, reaching 11.8 mg/g-DCW—surpassing levels observed under Condition A. Astaxanthin productivity reached 7.5 mg/L/day on day 7; however, this was not significantly different from that of Strain 1 cultivated under identical conditions (Figure 7c). This suggests that under light-saturating conditions, the benefit of *tkt* overexpression may be limited by other physiological constraints, such as reduced growth or downstream bottlenecks.

## Discussion

A comparison between the astaxanthin-producing strain (Strain 1) and the wild type revealed that the introduction of *crtZ* and *crtW* decreased the accumulation of β-carotene and zeaxanthin while increasing the levels of astaxanthin intermediates such as echinenone and adonirubin (Figure 2e), which is consistent with our previous findings.^17^ Unlike the previous study, in which *crtZ* and *crtW* were expressed from the pAQ plasmid, these genes were integrated into the genome in Strain 1. In general, plasmid-based expression results in a higher gene copy number and greater enzyme expression, whereas genomic integration offers increased genetic stability during prolonged cultivation.^37^ Indeed, Nozzi et al. (2017) reported that protein expression from the pAQ plasmid in *Synechococcus* sp. PCC 7002 was approximately an order of magnitude higher than that achieved via chromosomal integration.^38^ Nevertheless, astaxanthin accumulation in Strain 1 (6.63 mg/g-DCW) was approximately double the amount reported in our previous study using *Synechococcus* sp. PCC 7002.^17^ To elucidate the underlying reasons for this discrepancy, future studies should measure the plasmid copy number, gene expression levels, and protein abundance under identical cultivation conditions.

This study demonstrates a metabolomics-guided strategy for improving astaxanthin production in *Synechococcus* sp. PCC 7002. The *tkt*-overexpressing strain (Strain 5) achieved the highest astaxanthin content and productivity among the recombinant strains in this study, reaching 10.3 mg/g-DCW and 3.5 mg/L/day, respectively (Figure 5). TKT is a known metabolic engineering target within the Calvin cycle and has previously been used to enhance ethanol production in cyanobacteria.^30^ In contrast, other Calvin cycle enzymes—such as F/SBPase and ribulose-1,5-bisphosphate carboxylase/oxygenase (RuBisCO)—have more frequently been targeted to improve carbon fixation and/or biomass production in cyanobacteria.^30,31,39,40^ Under the conditions used in this study, overexpression of F/SBPase unexpectedly reduced the level of astaxanthin production. This result highlights that even within the Calvin cycle, it is difficult to predict which enzyme overexpression will enhance product yield. Metabolomics analysis provides a valuable means of identifying these productive targets in advance, enabling the more rational design of strain engineering strategies. In contrast, overexpression of *ispG* did not lead to an increase in astaxanthin content (Figure 5). Although the accumulation of MEcPP was alleviated—indicating that the IspG-catalyzed step was no longer limiting (Figure 6)—this relief of a metabolic bottleneck did not translate into improved astaxanthin production. This may be due to the emergence of a new downstream bottleneck or an imbalance in the metabolic flux. Similar findings have been reported in *E. coli*, where overexpression of *ispG* led to accumulation of the intermediate HMBPP, reduced cell growth, and decreased carotenoid production, unless the downstream enzyme *ispH* was coexpressed to balance the pathway. Coexpressing *ispG* with other genes located downstream of IspG, such as *ispH* and *idi,* in PCC 7002 is therefore a logical next step.^200^ Recently, Betterle et al. (2025) demonstrated that introducing *crtZ* and *crtW* into *Synechococcus* sp. PCC 11901, a close relative of PCC 7002, enabled astaxanthin production at a rate of 10 mg/L/day (Table 1).^41^ Given the metabolic similarities between these strains, the engineering strategies identified in this study are expected to be effective in further enhancing astaxanthin production in PCC 11901 as well. In addition, we acknowledge that comprehensive metabolomics of higher-producing derivatives, such as Strains 2 and 3, would likely reveal further network rewiring and help uncover additional engineering targets; this will be an important direction for future work.

While *dxs* or *crtE* individually improved specific metrics in our setting, their coexpression (Strain 7) yielded only a partial, late-stage gain and did not outperform the *tkt* strain (Figure S4). This illustrates a common feature of pathway engineering: single beneficial edits need not combine additively because flux control is distributed and gene interactions are epistatic across central carbon, isoprenoid precursors, and carotenoid tailoring steps. Consequently, identifying productive combinations typically requires a rational multitarget search rather than simple stacking—a point underscored by recent work where only specific multigene sets produced synergy, whereas other combinations were neutral or suboptimal.^42^ A broader combination scan will be required to map synergy vs antagonism and constitutes a key limitation and direction for future work.

Prior reports in *Synechocystis* PCC 6803 showed that *tktA* overexpression can cause a chlorotic phenotype under moderate to high light, likely by diverting triose-phosphate away from RuBP regeneration and increasing oxidative stress.^40,43^ In our *Synechococcus* PCC 7002 strain (Strain 5), by contrast, no pronounced chlorosis was observed: chlorophyll *a* was not broadly depleted, and biomass was only modestly reduced, while astaxanthin increased (Figures 5, S2, and S3). Although this difference might be attributed to host-specific carbon allocation/redox control (7002 vs 6803), future work will titrate *tkt* expression and coengineer RuBP regeneration to define the boundary between beneficial flux reinforcement and chlorosis.

When cultured under high-cell-density conditions (Condition B), Strain 5 exhibited increased astaxanthin productivity compared to the standard culture conditions (Condition A). However, under these high-density conditions, its productivity remained comparable to that of Strain 1. This suggests that the metabolic bottleneck may shift to other limiting factors, such as RuBisCO activity, ATP/NADPH regeneration, or CO_2_ supply, particularly under high light conditions where the photosynthetic apparatus is already operating near its capacity.^44^ Under such light-saturating conditions, further increases in the Calvin cycle flux via transketolase overexpression are unlikely to enhance overall carbon assimilation. These findings highlight the need for additional strain engineering and further optimization of cultivation parameters tailored to the physiological constraints of *Synechococcus* sp. PCC 7002.

## Materials and Methods

### Strain and Culture Conditions

*Synechococcus*sp. PCC 7002 strain and its derivatives used in this study are shown in Table S1. The strains were commonly cultured in MA2 medium (4.25 g/L NaNO_3_, 50 mg/L KH_2_PO_4_, 18 g/L NaCl, 5 g/L MgSO_4_·7H_2_O, 0.37 g/L CaCl_2_·2H_2_O, 0.6 g/L KCl, 32 mg/L Na_2_EDTA·2H_2_O, 8 mg/L FeCl_3_·6H_2_O, 34 mg/L H_3_BO_3_, 4.3 mg/L MnCl_2_·4H_2_O, 0.32 mg/L ZnCl_2_, 50 μg/L Na_2_MoO_4_·2H_2_O, 3.0 μg/L CuSO_4_·5H_2_O, 12 μg/L CoCl_2_·6H_2_O, 4.0 μg/L cobalamin, and 8.3 mM tris­(hydroxymethyl) aminomethane pH 8.2).^45^ White fluorescent lamps with peak wavelengths of ∼545 and ∼613 nm were used as the light source for cultivation. Transformants were precultured at 30 °C for 3 days under a light intensity of 100 μmol/m^2^/s. Cells were grown in 70 mL of MA2 medium containing 2% (v/v) CO_2_ and 40 μg/mL kanamycin, shaking at 100 rpm in a two-stage flask.^46^ After preculture, cells were inoculated into fresh MA2 medium to an initial OD_750_ of 0.1 and cultured at 2% (v/v) CO_2_, 100 μmol/m^2^/s of white light, 100 rpm, and 30 °C for 7 days. The culture condition was designated “Condition A”. For the high-cell-density cultivation, cyanobacteria cells were cultured in MAD2 medium (16.3 g/L NaNO_3_, 0.28 g/L KH_2_PO_4_, 18 g/L NaCl, 5 g/L MgSO_4_·7H_2_O, 0.37 g/L CaCl_2_·2H_2_O, 0.6 g/L KCl, 32 mg/L Na_2_EDTA·2H_2_O, 0.13 g/L FeCl_3_·6H_2_O, 2.86 mg/L H_3_BO_3_, 1.81 mg/L MnCl_2_·4H_2_O, 0.22 mg/L ZnSO_4_·7H_2_O, 1.26 mg/L Na_2_MoO_4_·2H_2_O, 0.08 mg/L CuSO_4_·5H_2_O, 40.3 μg/L CoCl_2_·6H_2_O, 12.2 μg/L cobalamin, and 8.6 mM tris­(hydroxymethyl) aminomethane, pH 8.2). White fluorescent lamps with peak wavelengths of ∼545 and ∼613 nm were used as the light source for cultivation. Transformants were precultured at 100 μmol/m^2^/s, 30 °C for 3 days in MA2 medium under 2% (v/v) CO_2_ with 40 μg/mL kanamycin, shaking at 100 rpm in two-stage flasks. After preculture, cells were inoculated into MAD2 medium to an OD_750_ of 0.5 and cultured overnight at 38 °C under 5% (v/v) CO_2_ and 150 μmol/m^2^/s white light, shaking at 150 rpm. Light intensity was increased to 500 μmol/m^2^/s after 1 day of culturing, and the culture was continued for the next 6 days. The culture condition was designated “Condition B”. Cell density in the culture was measured by OD_750_ and converted to dry cell weight (DCW) using a calibration curve established between OD_750_ and DCW.

### Construction of Recombinant Strains

*E. coli* DH5α was used for the construction of plasmids shown in Table S2. Target genes shown in Table S3 were amplified using KOD One PCR Master Mix/Blue (TOYOBO, Osaka, Japan) under standard PCR conditions by using primers listed in Table S4. The amplified fragments were assembled using In-Fusion Snap Assembly Master Mix (Takara Bio Inc., Shiga, Japan), and plasmids were constructed to express each gene of interest within cyanobacteria. Wild-type *Synechococcus* sp. PCC 7002 was transformed with the respective plasmids to obtain the desired recombinant strains. Cells cultured in A2 medium to an OD_750_ of 1.0 were mixed with the plasmid (1 μg per 100 μL of culture). The mixture was spread onto a 0.45 μm nitrocellulose membrane filter (Millipore, Billerica, MA) placed on MA2 agar plates and incubated under white light at 30 °C for 3 days. The filter was then transferred to another MA2 agar plate containing antibiotics, and single colonies were repeatedly isolated until successful recombination of the target gene into the *Synechococcus* sp. PCC 7002 genome was confirmed using the primer sets shown in Table S4.

### Metabolome Analysis

Metabolome samples were prepared as described in previous studies. A 1:4 ratio of culture (containing 5 mg DCW) to prechilled (−30 °C) 32.5% (v/v) methanol was used. After centrifugation at 8000 *g* for 5 min at −4 °C, the supernatant was removed. The cells were washed with 20 mM ammonium carbonate (pH 8.7), centrifuged at 8000 *g* for 5 min at −4 °C, and the washing solution was discarded. The cells were suspended in 1 mL of methanol containing 37.5 μM methionine sulfone and 37.5 μM piperazine-1,4-bis­(2-ethanesulfonic acid) as internal standards for MS analysis. To 0.5 mL of the suspension, 0.2 mL of ice-cold water and 0.5 mL of chloroform were added. After vortexing for 30 s, the sample was centrifuged at 14,000 *g* for 5 min at 4 °C. The upper aqueous phase (500 μL) was concentrated using a 3 kDa cutoff membrane (Millipore) and then vacuum-dried. The dried extracts were dissolved in Milli-Q water and analyzed using a capillary electrophoresis-mass spectrometry (CE-MS) system (Agilent Technologies, Palo Alto, CA) as described in the literature.^46^

### Pigment Analysis

The analysis of the recovered cells was performed by optimizing a previously described method.^17^ Cultures containing 5 mg of DCW were centrifuged at 8000 × g for 3 min at 4 °C, and the cells were washed with 20 mM ammonium carbonate (pH 8.7). After washing, 0.5 mL of methanol was added, and the cells were disrupted using a Multi-Beads Shocker (MB2000, Yasui Kikai, Osaka, Japan) at 2700 rpm for 60 min at 4 °C. After disruption, 0.5 mL of methanol, 0.3 mL of chloroform, and 0.1 mL of water were added and vortexed. The mixture was centrifuged at 14000*g* for 5 min at 4 °C, and 0.98 mL of the upper phase was collected. After 0.44 mL of water was added and vortexed, the sample was centrifuged again at 14,000 *g* for 5 min at 4 °C. For each 50 μL of the lower phase, 450 μL of 8:2 (v/v) acetonitrile containing 0.1 mM trans-β-apo-8’-carotenal as the internal standard was added for analysis. Carotenoids and chlorophyll a were quantified using an Acquity ultraperformance liquid chromatography (UPLC) system (Waters Corporation, Milford, MA) with a photodiode array (PDA) detector, according to the previously reported method.^47^

## Supplementary Material





## Notes

Pigment data other than astaxanthin; effect of coexpressing *dxs* and *crtE*; strains, plasmids, and primers used in this study (PDF)
Nucleotide sequences used in the study (XLSX)
