# Supplementary material for: Astaxanthin Overproduction Enhanced by Metabolomics-Guided Rational Metabolic Engineering in Synechococcus sp. PCC 7002
Source: ACS Synth Biol. 2025 Nov 10;14(11):4467–77. doi: 10.1021/acssynbio.5c00490 (PMC12645575; doi:10.1021/acssynbio.5c00490)
Supplement: Supplementary file 1 [file sb5c00490_si_001.pdf]

Supplementary information

Astaxanthin overproduction enhanced by metabolomics-guided rational metabolic engineering in *Synechococcus* sp. PCC 7002

Kousuke Ida[#1], Kenya Tanaka[#2,3,4], Yuichi Kato[5], Nobuaki Koike[1], Yoji Horie[1], Mami Matsuda[3], Hisashi Yasueda[3,8], Akihiko Kondo[2,3,6,7], Tomohisa Hasunuma[2,3,6*]

# equally contributed

[1] Kawasaki Frontience R&D Center, Toagosei Co., Ltd., 3-25-40 Tonomachi, Kawasaki, Kawasaki, Kanagawa 210-0821, Japan

[2] Engineering Biology Research Center, Kobe University, 1-1 Rokkodai, Nada, Kobe 657-8501, Japan

[3] Graduate School of Science, Innovation and Technology, Kobe University, 1-1 Rokkodai, Nada, Kobe 657-8501, Japan

[4] Research Center for Solar Energy Chemistry, Graduate School of Engineering Science, The University of Osaka, Toyonaka, Osaka 560-8531, Japan

[5] Department of Biotechnology, Toyama Prefectural University, 5180 Kurokawa, Imizu, Toyama 939-0398, Japan

[6] RIKEN Center for Sustainable Resource Science, 1-7-22 Suehiro, Tsurumi, Yokohama, Kanagawa 230-0045, Japan

[7] Department of Chemical Science and Engineering, Graduate School of Engineering, Kobe University, 1-1 Rokkodai, Nada, Kobe 657-8501, Japan

[8] Research and Development Center for Precision Medicine, University of Tsukuba, 1-2 Kasuga, Tsukuba-shi, Ibaraki, 305-8550, Japan

(*Corresponding Author: Tomohisa Hasunuma, hasunuma@port.kobe-u.ac.jp)

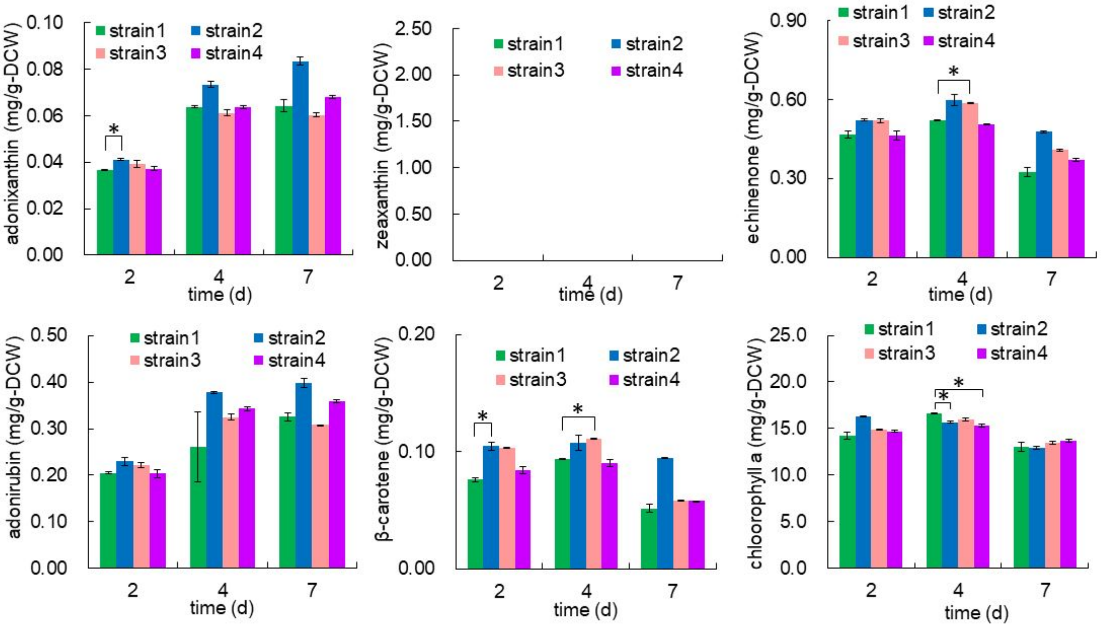

Supplementary Fig. S1. Time courses of adonixanthin, adonirubin, echinenone, β-carotene, zeaxanthin, and chlorophyll a (mg/g-DCW) for Strains 1–4 under Condition A. Mean ± SE; n = 2; occasional pairwise significance is indicated by asterisks in the panels (* $p < 0.05$).

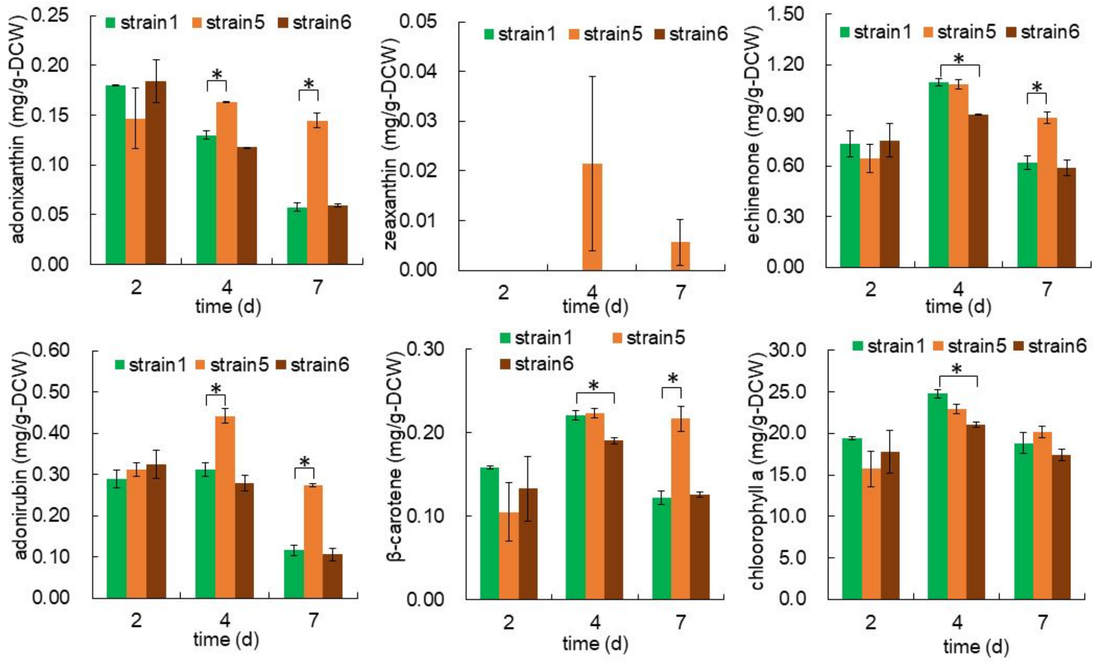

Supplementary Fig. S2. Time courses of adonixanthin, adonirubin, echinenone, β-carotene, zeaxanthin, and chlorophyll a (mg/g-DCW) for Strains 1,5, and 6 under Condition A. Mean ± SE; n = 3; Pairwise significance is indicated by asterisks in the panels (* $p < 0.05$).

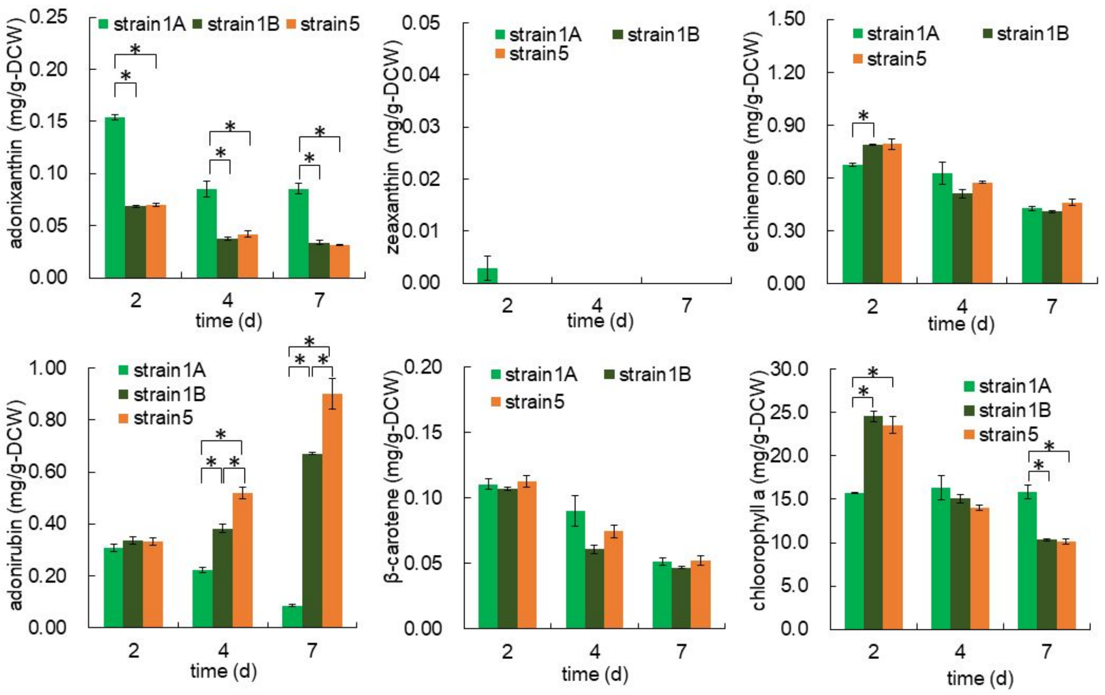

Supplementary Fig. S3. Time courses of adonixanthin, adonirubin, echinenone, β-carotene, zeaxanthin, and chlorophyll a (mg/g-DCW) for Strains 1 (under Condition A and B) and 5 (under condition B). Mean ± SE; n = 3; Pairwise significance is indicated by asterisks in the panels (* $p < 0.05$).

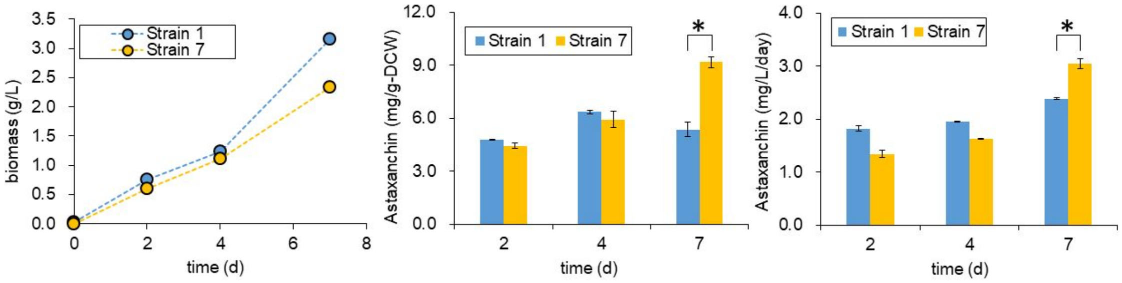

Supplementary Fig. S4. Additive effect of co-expressing *dxs* and *crtE*. (a) Time course of biomass under Condition A. (b) Cellular astaxanthin content (mg/g-DCW). (c) Volumetric astaxanthin productivity (mg/L/day). Blue, Strain 1; yellow, Strain 7. Values are mean ± SE (n = 3). Statistical significance at each time point was assessed by unpaired t-test (* $p < 0.05$).

Table S1. Cyanobacteria strains constructed in this study

Strain name	Description
WT	Wild-type *Synechococcus* sp. PCC 7002
Strain 1	WT, glpK::P_{psbA2}-crtZ-crtW, Kan^r
Strain 2	Strain 1, acsA::P_{psbA2}-dxs, Cm^r
Strain 3	Strain 1, A1202::P_{psbA2}-crtE, Gm^r
Strain 4	Strain 1, A0936::P_{rbcL}-fbp/sbp, Sp^r
Strain 5	Strain 1, acsA::P_{psbA2}-tkt, Cm^r
Strain 6	Strain 1, A0026::P_{psbA2}-ispG, Amp^r
Strain 7	Strain 2, A1202::P_{psbA2}-crtE, Gm^r

Table S2. Plasmids used in this study

Plasmid name	Description
pSKpsbA2-glpK-crtZW	glpK::P_{psbA2}-crtZ-crtW, Kan^r
pSCpsbA2-acsA-dxs	acsA::P_{psbA2}-dxs, Cm^r
pSGpsbA2-A1202-crtE	A1202::P_{psbA2}-crtE, Gm^r
pSSrbcL-A0936-fbp/sbp	A0936::P_{rbcL}-fbp/sbp, Sp^r
pSCpsbA2-acsA-tkt	acsA::P_{psbA2}-tkt, Cm^r
pSApsbA2-A0026-ispG	A0026::P_{psbA2}-ispG, Amp^r

Table S4. Primers used in this study

Primer	Sequence
glpK start-F1	GTGAATTCGAGCTCGGTACCTGAAGCGATTGGCTATGATCTACCAAAG
glpK start-R1	TGGCAATTCCGGCTCGAGTTTTTAAATGGGTTAAATTAGGTC
glpK stop-F1	CTAAGTGGGGAAACGCGTTTACTGCTCCATGACCAACATTATTCCC
glpK stop-R1	CATGATTACGCCAAGCTTGAAACGAGATTATCTAAAACAGAAGCATGG
crtZ-crtW-F1	GAGGACTGACCTAGCATATGGCTTGGCTTACTTGGATCGC
crtZ-crtW-R1	GTAATTGCCAAAACTGTAACCTGCAGGTCGACTCAAGACT
acsAA-F1	GTACGGTACCATGATCATCGGGGAATGCTCTTGATTC
acsAA-R1	TCGACTCGAGTCGTGGGATTTATTTCACCCCATTGTC
acsAB-F1	TAAGTGGGGAAACGCGTGTCTTAATGTATGAAGGCGCACCC
acsAB-R1	ATGATTACGCCAAGCTTCCTCTGGACATCTCCCTCAAGG
dxs-F1	GGACTGACCTAGCATATGAACGAACTACCAGGTACCAGCGATAC
dxs-R1	AACTGTAACCTGCAGTTAGACTTCAATCGGCACATCGACC
A1202_A1203-Fw1	CCATGATTACGAATTCTGGGGGCTTGTTGAAGATTTACTTTAGG
A1202_A1203-Rv1	CGGACATCAGCGCTCAAGTCTTAATTTATGGGCATCTCC
A1202_A1203-Fw2	CCTTGTGATGATCACTACCG
A1202_A1203-Rv2	GGCCAGTGCCAAGCTTTTTGAGGTAATTTTCGTTAATTCTAACC
crtE-F1	GAATTATAACCCATATGGTAGTTGCAGACG
crtE-R1	TAACCTGCAGGTCGACTTAGTTTTTACGGTT
fbp/sbp-F1	GGAGGACTGACCTAGGGATCCATGGAAAGCACCCTCGGTTTAGAAATTATTG
fbp/sbp-R1	AACTGTAACCTGCAGGTCGACTCTAGACTAGAGTTGAAT

	ATTTTGGGGGTTGTCCC
SpecR-InFusion-F1	TTAAAAACTGAGTAACTCGAGGGGGTCTGACGCTCAGTG GAAC
SpecR-InFusion-R1	GACTTTCTAATTAGAGCGGCCGCGTGGCACTTTTCGGGGA AATGTG
tkt-F1	ACCCATATGACCGTTGCAACCCAATCTATAGATCA
tkt-R1	GTAACCTGCAGGTCGACTTAACCTAGTACTGTCTCAG
ispG-F1	TAAGGAATTATAACCATATGCAAGCTGTAGAGCGTCCTG TGCAAACGACC
ispG-R1	GCCAAAACTGTAACCTGCAGGTCGACTATTGGGGCGGAT CAA
ispG-vector-F1	CTGCAGGTTACAGTTTTGGCAATTACTAAAAAACTGAC
ispG-vector-R1	AAGCATTGGTAACTAGTTTATCTACACAGTGATCATCA
PpsbA2-F1	GCGCTCGAGTCATTATTTCATCTCCA
PpsbA2-R1	CATATGGTTATAATTCCTTATGTATTTGTCGATGTTCAGAT TG
AmpR-F1	TAAACTAGTTACCAATGCTTAATCAGTGAGGCACCTATCT CAGCG
AmpR-R1	TGAAATAATGACTCGAGCGCGGAACCCCTATTTG
